# Biomarker guided combination strategies and perioperative integration for immune cold microsatellite stable colorectal cancer

**DOI:** 10.1007/s12672-026-04575-3

**Published:** 2026-02-10

**Authors:** Nan Yao, Wenqiang Li, Ning Duan, Fuzhou Han, Guoyong Yu, Jun Qu

**Affiliations:** 1 Department of General Surgery, Aerospace Center Hospital, Yuquan Road 15, Haidian District, Beijing, 100049 China; 2 Department of Nephrology, Beijing University of Chinese Medicine Affiliated Dongzhimen Hospital, Beijing, 100007 China; 3 Department of Nephrology, Beijing Dongzhimen Hospital, East 4th North Street 279, Dongcheng District, Beijing, 100007 China

**Keywords:** Colorectal cancer, MSS, pMMR, Immunotherapy, Combination therapy, Anti-VEGF, Radiotherapy, Dual checkpoint, EGFR, Biomarkers, CtDNA, Oligometastatic, Peri-operative

## Abstract

**Background:**

Most colorectal cancers (CRC) are microsatellite stable (MSS) and show little benefit from single-agent immune checkpoint inhibition (ICI). A rapidly expanding body of work now focuses on combination strategies to remodel the tumor microenvironment (TME) and convert immune-“cold” MSS disease into an immune-responsive state.

**Main body:**

We synthesize (i) biological liabilities that underpin MSS immune resistance; (ii) clinical evidence across key combination classes—anti-angiogenic/VEGFR + ICI (randomized AtezoTRIBE suggests a progression-free survival benefit when atezolizumab is added to FOLFOXIRI + bevacizumab), multikinase TKI + PD-1/PD-L1 (mixed activity and geographic/site heterogeneity; LEAP-017 was negative for overall survival), dual-checkpoint or next-generation CTLA-4 backbones (phase-1 botensilimab + balstilimab with durable responses in heavily pretreated MSS mCRC), EGFR- or HER2-targeted plus ICI (signals in molecularly defined subsets; e.g., CAVE rechallenge), and RT/ablation + ICI (biologically compelling but heterogeneous outcomes in pMMR-dominant settings). We emphasize biomarker-anchored selection—topology of disease (especially active liver metastases), inflammatory gene programs, and ctDNA kinetics—and propose peri-operative/oligometastatic integration frameworks relevant to surgical teams. Conclusions: Converting MSS CRC from cold to hot appears feasible in biologically selected contexts and with rational sequences. The next wave should stratify by liver involvement, embed ctDNA-anchored endpoints, and leverage surgery-embedded window studies to verify on-treatment immune conditioning.

## Introduction

Immune checkpoint blockade (ICB) is transformative in MSI-H/dMMR CRC, yet > 85% of CRC are MSS/pMMR and have minimal responses to PD-(L)1 or CTLA-4 monotherapy [1, 2]. In MSS disease, multiple layers of immune exclusion converge: low neoantigenicity and defective cross-presentation limit priming; WNT/β-catenin signaling, TGF-β–rich stromal programs (often CAF/CMS4-skewed), and aberrant vasculature collectively suppress dendritic cell recruitment and T-cell trafficking [3–6]. VEGF-driven endothelial anergy (reduced ICAM-1/VCAM-1), hypoxia/HIF-1α, and perivascular fibrosis further gatekeep lymphocyte entry [5]. The myeloid compartment is dominant and suppressive—M2-like TAMs, MDSCs, and TANs elaborate IL-10, TGF-β, arginase and ROS, while chemokine axes (e.g., CXCL8–CXCR1/2) perpetuate exclusion [7, 8]. Metabolic and epigenetic brakes—adenosine–A2A, IDO1/tryptophan catabolism, lactate/acidic pH, and repressive chromatin states—dampen type-I/II interferon programs and cytotoxic effector function [9–11].

Clinically, hepatic metastases impose a uniquely tolerogenic niche: antigen is filtered through Kupffer cells and sinusoidal endothelial cells, with antigen-presenting cell skew and PD-L1/galectin-9 up-regulation that delete or exhaust circulating tumor-specific T cells and blunt systemic ICI effects; by contrast, extrahepatic-dominant disease shows relatively higher immune responsiveness [12, 13].

While liver metastases can create a tolerogenic niche through macrophage polarization, antigen sequestration/deletion, and systemic T-cell dysfunction, the presence of hepatic disease in clinical cohorts should also be interpreted with epidemiologic nuance, as it is an aggregate marker that often correlates with higher tumor burden, more aggressive disease kinetics, and tumor-intrinsic programs (e.g., CMS2/CMS4 under CMS subtyping) that themselves shape outcomes. Therefore, an observed “liver effect” in immunotherapy-treated MSS CRC may reflect a true immunologic barrier, a prognostic confounder, or both. To reduce conflation of metastatic site with tumor biology, we reorganized the evidence synthesis into (i) mechanistic/translational data supporting liver-driven systemic immune suppression, (ii) clinical association studies, and (iii) studies incorporating adjustment or stratification by tumor burden surrogates and tumor-intrinsic features where available. Importantly, primary tumor sidedness (right vs. left) correlates with molecular background and metastatic patterns and should be considered when interpreting hepatic metastasis as a modifier of immunotherapy benefit. Future trials and real-world analyses should prespecify interaction testing (liver vs. non-liver; sidedness; molecular subtype) and incorporate tumor burden metrics (e.g., number/volume of metastatic lesions, LDH) to better disentangle barrier versus confounding effects.

These insights motivate combination regimens that (i) normalize vasculature (anti-VEGF/VEGFR) to open trafficking windows [14]; (ii) reprogram myeloid networks (e.g., CSF1R, CXCR1/2, TGF-β traps) to restore antigen presentation [6, 7, 15, 16]; (iii) increase antigen release/visibility (RT/SABR, oncolytic or EGFR/HER2-directed ADCC, bispecifics such as CEA-TCB) [17–19]; and (iv) relieve metabolic/epigenetic brakes (A2A antagonists, IDO-pathway, DNMT/HDAC modulators)—ideally converting MSS tumors into sustained, IFN-rich inflamed TMEs [9–11]. Embedding biomarker-anchored selection (active liver involvement, T-cell-inflamed signatures, and on-treatment ctDNA kinetics) and sequence timing (e.g., short VEGF-normalization windows before ICI) will be critical to translate this biology into durable clinical benefit [12, 14].

### Scope and search

We conducted a prespecified narrative review of biomarker-guided combination strategies to overcome resistance in MSS/pMMR colorectal cancer. We searched PubMed/MEDLINE, Embase, Web of Science, Cochrane CENTRAL, ClinicalTrials.gov, and ASCO/ESMO/AACR abstracts from 1 Jan 2015 to 31 Oct 2025. Queries combined CRC + MSS/pMMR + immunotherapy (PD-1/PD-L1/CTLA-4) with major partners (anti-VEGF/VEGFR, EGFR/ADCC, liver-directed RT/ablation, epigenetic/metabolic, microbiome); key trial/drug names (e.g., AtezoTRIBE, LEAP-017, CAVE, botensilimab+balstilimab, REGONIVO/REN-series) were added to capture signal-defining studies. Inclusion: prospective trials, peri-operative/window studies, or comparative cohorts that enrolled MSS populations (or extractable MSS subgroups) and reported ORR/PFS/OS and/or human correlatives (e.g., ctDNA dynamics). Exclusion: MSI-H/dMMR-only cohorts, case reports/very small series, and purely preclinical work. Two reviewers screened records with consensus resolution. Evidence is summarized by design tier—A: randomized; B: prospective non-randomized; C: retrospective comparative with adjustment; D: exploratory signals/correlatives—with mixed-population trials labeled and reported by their MSS-specific results.

### Mechanisms of resistance in MSS CRC

#### Antigenicity and presentation

Most MSS tumors carry low neoantigen loads and often harbor defects in the antigen-presentation axis (e.g., down-modulated HLA class I, B2M loss, impaired IFN-γ/JAK–STAT signaling) [20–24]. Oncogenic programs like WNT/β-catenin suppress dendritic-cell recruitment (via reduced CCL4/CCL5 and CXCL9/10) and blunt priming in tumor-draining nodes, yielding a T-cell–excluded phenotype even when PD-1/PD-L1 is blocked [25, 26].

#### Vascular abnormality and hypoxia

VEGF-driven “endothelial anergy” lowers ICAM-1/VCAM-1 and tightens the endothelial barrier, throttling lymphocyte trafficking [5, 27]. Hypoxia and HIF-1α foster acidosis and perivascular fibrosis, further limiting infiltration. Short-lived “vessel normalization” with anti-VEGF/VEGFR therapy can transiently reopen the gate for immune recruitment—most useful when synchronized with T-cell–activating strategies (ICB, vaccines, RT/ablation) [27–29].

#### Myeloid-dominant suppression

M2-skewed TAMs, granulocytic/monocytic MDSCs, and TANs accumulate under CXCL8–CXCR1/2, CSF1/CSF1R, and GM-CSF axes. They secrete IL-10, TGF-β, ARG1 and ROS, quenching CD8⁺ cytotoxicity and dendritic cross-presentation while siphoning nutrients (arginine, cysteine). Targetable hubs include CXCR1/2, CSF1R, and TAM-reprogramming approaches (CD40 agonism, PI3K-γ inhibition) [8, 15, 30–32].

#### Metabolic/epigenetic brakes

Tumor and stromal cells generate lactate (LDHA) and adenosine (CD39/CD73), activating A2A signaling and acidifying the TME—both dampen TCR signaling and IFN programs [33–36]. Tryptophan catabolism (IDO1/TDO2→kynurenine→AhR) reinforces exhaustion [37, 38], and repressive chromatin states (DNMT/HDAC/PRC2 activity) silence antigen-presentation and Th1 chemokines. Combinatorial tactics include A2A antagonists, CD73 blockade, metabolic buffering, and epigenetic “priming” (DNMT/HDAC inhibitors) to restore antigenicity and inflamed gene signatures [39–43].

#### Anatomical context

Active liver metastases create systemic tolerance: hepatic myeloid cells and LSECs cross-present antigen in a tolerogenic fashion, delete/disable circulating CTLs, and skew cytokine milieus toward IL-10/TGF-β [44–46]. However, in clinical cohorts, liver involvement may also proxy higher tumor burden and tumor-intrinsic programs; therefore, observed associations likely reflect both immunologic barrier effects and prognostic confounding, motivating prespecified interaction analyses (liver vs. non-liver; sidedness; molecular subtype) and inclusion of burden metrics (e.g., LDH, lesion number/volume). Clinically, multiple VEGFR-IO and dual-IO regimens show attenuated benefit when liver disease is untreated, whereas responses concentrate in non-liver-metastatic or liver-controlled cohorts—supporting aggressive liver-directed therapy (resection, ablation, radiation, or effective hepatic arterial strategies) to “unlock” systemic ICI [47–50].

**Fig. 1:**
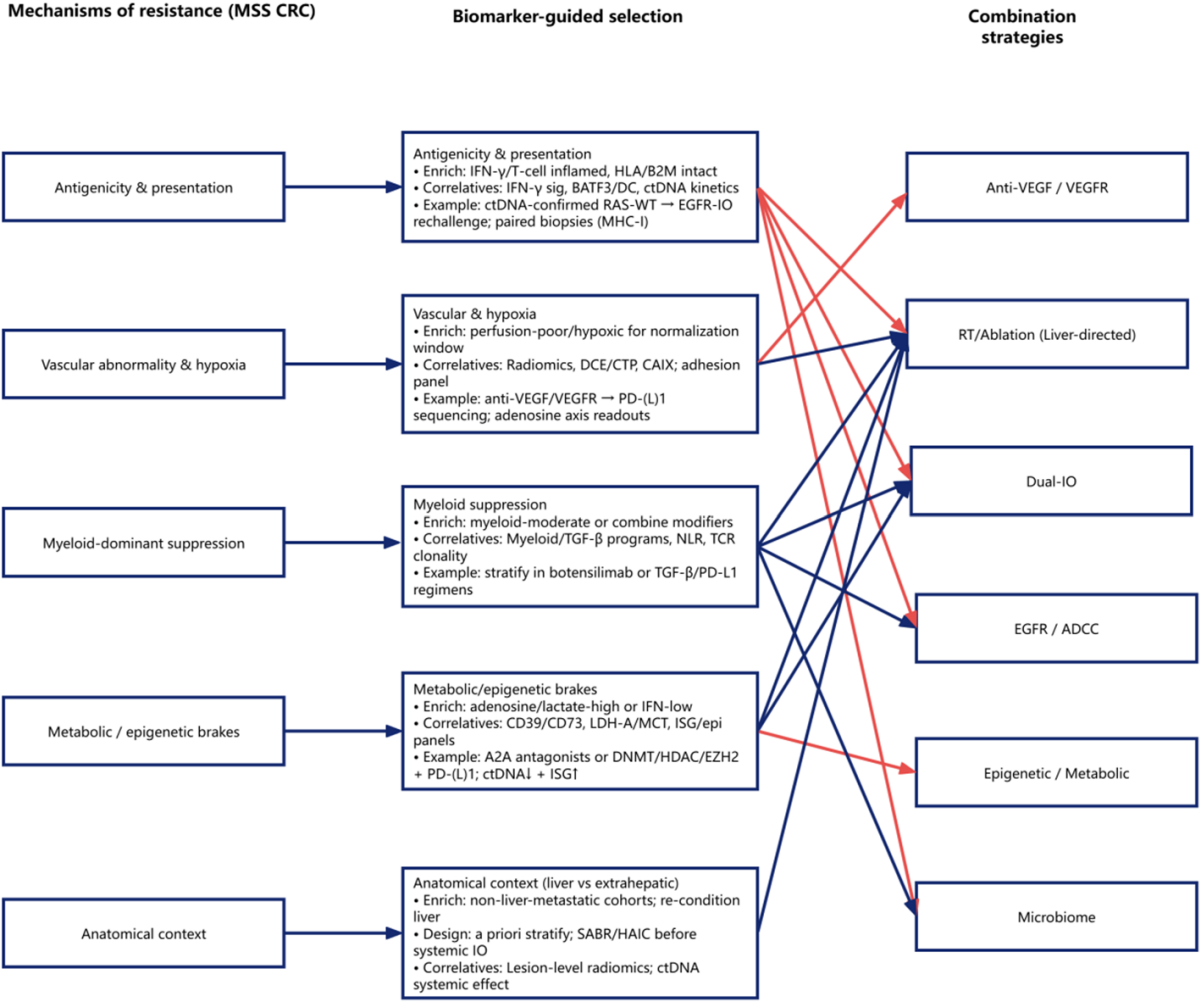
Mechanism-based, biomarker-guided combination strategies for immune-cold MSS colorectal cancer. Conceptual framework linking key resistance mechanisms in MSS colorectal cancer (left) to biomarker-guided selection/monitoring domains (middle) and corresponding combination strategy modules (right).* MSS* microsatellite stable,* CRC* colorectal cancer,* ctDNA* circulating tumor DNA,* EGFR* epidermal growth factor receptor,* VEGF* vascular endothelial growth factor,* VEGFR* vascular endothelial growth factor receptor,* RT* radiotherapy,* ADCC* antibody-dependent cellular cytotoxicity,* ICI* immune checkpoint inhibitor,* IO* immuno-oncology,* IFN-γ* interferon gamma,* HLA* human leukocyte antigen,* B2M* beta-2 microglobulin,* MHC-I* major histocompatibility complex class I,* DCE* dynamic contrast-enhanced,* CTP* computed tomography perfusion,* CAIX* carbonic anhydrase IX,* TGF-β* transforming growth factor beta,* NLR* neutrophil-to-lymphocyte ratio,* TCR* T-cell receptor,* LDH-A* lactate dehydrogenase A,* MCT* monocarboxylate transporter,* ISG* interferon-stimulated genes

A mechanism-to-biomarker-to-therapy framework is summarized in Fig. 1.

### Evidence across combination classes

Key representative trials across combination classes and their biomarker roles are summarized in Table 1.

**Table 1:** Representative clinical studies of combination strategies for MSS/pMMR metastatic colorectal cancer

Strategy	Study	author	References	Biomarker role	Results
Anti-angiogenic + ICI	FOLFOXIRI + Bev±Atezo vs. FOLFOXIRI + Bev	Carlotta Antoniotti	Antoniotti et al. AtezoTRIBE. *Lancet Oncol.* 2022;23:876–887	Prognostic	PFS improved: 13.1 vs. 11.5 months; HR = 0.69 *p* = 0.012
Radiotherapy/ablation + ICI	TNT(FOLFOX→CRT)±Pembro vs. TNT(FOLFOX→CRT)	Osama E. Rahma	Rahma et al. NRG-GI002 (pembro arm). *JAMA Oncol.* 2021;7:1225–1230.	Monitoring	NAR 11.53 vs. 14.08, *p* = 0.26; pCR 31.9% vs. 29.4%, *p* = 0.75; G3–4 AEs 48.2% vs. 37.3%
Dual-checkpoint or next-generation IO backbones	Botensilimab+Balstilimab	Andrea J Bullock	Bullock et al. BOT + BAL in MSS mCRC (Ph1). *Nat Med.* 2024;30:2558–2567.	Prognostic	ORR 17%;mPFS 3.5 months༛mOS 20.9 months
EGFR/HER2-targeted + ICI	Cetuximab + Avelumab	Erika Martinelli	Martinelli et al. CAVE trial. *JAMA Oncol.* 2021;7:1529–1535.	Predictive	mOS 11.6 months; mPFS 3.6 months༛ORR 7.8% ༛DCR 65%
Epigenetic/metabolic re-programming + ICI	Entinostat+ Pembrolizumab	Nilofer Saba Azad	Azad et al. ENCORE-601 (enti+pembro) in MSS mCRC. *JCO* 2018;36(15_suppl):3557.	Monitoring	RR 6%(1/16), mPFS 86days, SD 9/16
Microbiome and barrier modulation + ICI	Tislelizumab + Fruquintinib + FMT	Wensi Zhao	Zhao et al. RENMIN-215 update. *AJCR* 2024;14:5351–5364.	Monitoring	ORR 20%(4/20);DCR 95%༛mOS 13.7 months

Selected trials and cohorts are summarized by strategy class, regimen, and key efficacy outcomes. ICI, immune checkpoint inhibitor; IO, immuno-oncology; FOLFOXIRI, fluorouracil (5-FU), leucovorin, oxaliplatin, and irinotecan; FOLFOX, fluorouracil (5-FU), leucovorin, and oxaliplatin; TNT, total neoadjuvant therapy; CRT, chemoradiotherapy; Bev, bevacizumab; Atezo, atezolizumab; Pembro, pembrolizumab; EGFR, epidermal growth factor receptor; HER2, human epidermal growth factor receptor 2; FMT, fecal microbiota transplantation; PFS, progression-free survival; OS, overall survival; mPFS, median progression-free survival; mOS, median overall survival; ORR, objective response rate; DCR, disease control rate; RR, response rate; SD, stable disease; HR, hazard ratio; NAR, neoadjuvant rectal score; pCR, pathological complete response; AEs, adverse events; G, grade.

#### Anti-angiogenic (anti-VEGF/VEGFR) + ICI

Rationale. Vascular normalization increases antigen trafficking and T-cell infiltration; VEGF blockade also reduces Treg/MDSC recruitment [14, 27, 51]. AtezoTRIBE (first-line, randomized Phase II). Adding atezolizumab to FOLFOXIRI + bevacizumab in previously untreated mCRC improved PFS: 13.1 vs. 11.5 months; HR 0.69 (80% CI 0.56–0.85). Safety was manageable with more neutropenia/diarrhea but no new signals. This provides proof-of-concept for chemo + anti-VEGF + PD-L1 intensification up-front, in an unselected population with majority MSS [52]. TKI + PD-1 in refractory MSS. The US regorafenib + nivolumab phase-2 trial treated 70 of 94 enrolled; ORR 7%, PFS 1.8 months [47]; all responses occurred in patients without liver metastases.

Geographic and site-of-disease heterogeneity has been repeatedly observed, with poorer outcomes in liver-dominant disease and modest benefit overall.

LEAP-017 (lenvatinib + pembrolizumab, randomized Phase III). In 480 previously treated pMMR/MSS mCRC patients, OS 9.8 vs. 9.3 months; HR 0.83 (95% CI 0.68–1.02) did not meet the prespecified significance threshold, confirming no statistically significant OS improvement versus regorafenib or trifluridine/tipiracil. Safety was as expected for the class [53].

#### Radiotherapy/ablation (RT/SABR) + ICI

Rationale. RT enhances antigen release, MHC-I expression and type-I IFN signaling, potentially driving abscopal effects with PD-(L)1. Thermal ablation/SABR may achieve similar immunogenic cell death [54].

Clinical picture. In locally advanced rectal cancer (LARC) enriched for pMMR/MSS, adding pembrolizumab to TNT/CRT did not improve the neoadjuvant rectal (NAR) score in a randomized phase-2 trial, despite acceptable safety—underscoring that pMMR tumors require deeper biological selection or different sequencing [55].

In oligometastatic disease, early studies show feasibility and occasional systemic responses; however, dose/fractionation and IO timing (pre- vs. post-RT) remain unresolved in MSS-focused randomized data [56, 57].

#### Dual-checkpoint or next-generation IO backbones

Rationale. CTLA-4 (priming) + PD-1 (effector rescue) increases T-cell breadth; Fc-engineered CTLA-4 (e.g., botensilimab) may enhance intratumoral Treg depletion and myeloid re-programming.

Phase-1 botensilimab + balstilimab. In 148 heavily pretreated MSS mCRC patients (MSS expansion; 101 response-evaluable), ORR 17% (95% CI 10–26), DCR 61%, median PFS 3.5 months, and durable responses were reported with manageable immune toxicity, with activity enriched in patients without active liver metastases [58, 59].

#### EGFR/HER2-targeted + ICI

Rationale. Cetuximab (IgG1) can trigger ADCC, facilitate antigen cross-presentation and prime for PD-L1 blockade; HER2-targeted agents may also engage immunity.

CAVE/CAVE-2 (EGFR rechallenge). In RAS wild-type mCRC previously benefiting from anti-EGFR, cetuximab + avelumab showed disease control and signs of activity as a rechallenge strategy (*n* ≈ 77 in CAVE). ctDNA-confirmed RAS/BRAF WT status appears to enrich benefit.

Randomized MSS-specific confirmation is needed [60, 61].

#### Epigenetic/metabolic re-programming + ICI

DNMT/HDAC inhibitors, adenosine A2A antagonists, tryptophan/IDO pathway inhibitors, and lactate transport blockade are being tested to lift transcriptional/metabolic brakes and restore interferon signaling. While the mechanistic rationale is strong, definitive randomized signals in MSS CRC remain pending; rational triplets (e.g., VEGF + epigenetic modulator + PD-1) in adaptive platforms may be required to identify synergistic windows [42, 62–65].

#### Microbiome and barrier modulation + ICI

Gut microbiota shape APC programming and T-cell tone. Early MSS CRC experiences using FMT or targeted pro-/post-biotics are small but feasible with rigorous donor safety. Prospective trials integrating shotgun metagenomics/metabolomics with IO readouts (and ctDNA) are a high-yield path forward [66–68].

### Biomarker-guided selection

Prognostic, predictive, and response-monitoring biomarkers In MSS CRC, many candidate biomarkers are discussed under the umbrella of “biomarker-guided selection,” yet their evidentiary roles differ. We therefore distinguish: (i) prognostic biomarkers that correlate with outcomes irrespective of treatment, (ii) predictive biomarkers that modify the likelihood of benefit from a specific therapeutic class, and (iii) pharmacodynamic/response-monitoring biomarkers that capture early on-treatment biological response. For example, ctDNA kinetics and clearance are robust markers of tumor burden dynamics and treatment response, and can serve as early readouts to support continuation, escalation, or mechanism switching; however, ctDNA clearance alone does not inherently specify whether a VEGF-, EGFR-, or RT-anchored combination is the optimal initial strategy. Where predictive evidence is limited in MSS CRC immunotherapy combinations, we explicitly label biomarkers as hypothesis-generating and prioritize trial designs incorporating prospective stratification and interaction testing.

Disease topology (liver vs. extrahepatic). Across regorafenib + nivolumab and RIN (regorafenib + ipilimumab + nivolumab), responses were essentially absent with active liver metastases and concentrated in non-liver-metastatic disease [47, 69]. This consistent pattern argues for a-priori stratification by liver involvement in trial design and for liver-directed “re-conditioning” strategies when feasible [49, 70].

Transcriptional inflammation. T-cell-inflamed IFN-γ signatures and myeloid/TGF-β programs may define “heatable” niches vs. profoundly excluded tumors; embedding tissue and liquid-biopsy correlatives is essential [71, 72].

ctDNA kinetics. On-treatment ctDNA decline/clearance provides an early, mechanism-anchored readout that may predict durable benefit and enable adaptive dose/sequence optimization across platforms [73, 74].

Oncogenic drivers and CMS. RAS/BRAF status, CMS subtypes and WNT-β-catenin activation correlate with exclusion; such features should guide inclusion/enrichment or rational combination choice (e.g., EGFR-IO rechallenge in ctDNA-confirmed RAS-WT) [75, 76].

Radiomics and peripheral immunology. Baseline and delta-radiomics capturing perfusion/texture and peripheral TCR clonality/myeloid ratios can complement ctDNA in MSS-focused trials [77].

### Surgical and locoregional integration (peri-operative and oligometastatic care)

Curative intent for oligometastatic MSS CRC still hinges on resection/ablation/SABR. Rational IO integration for surgeons includes:

Timing.

Neoadjuvant priming (e.g., short “vascular normalization” window with anti-VEGF ± PD-1) to improve T-cell trafficking before resection/ablation [78].

Post-ablation/SABR consolidation PD-(L)1 to exploit antigen burst and potential systemic immunity [79].

Peri-operative immunology. Surgical stress can transiently suppress anti-tumor immunity; window-of-opportunity designs should sample PBMCs, TME biopsies, and ctDNA (pre-op; POD1-3; POD7-14; 6–8 weeks) to document whether we are truly “heating” tumors [80, 81].

Anatomical site strategy. In liver-dominant disease, consider hepatic re-conditioning (e.g., liver-directed RT/SABR in trials) before systemic dual-IO; extrahepatic-dominant patients may preferentially enter next-gen CTLA-4 + PD-1 or VEGFR-IO arms [70, 82].

Multidisciplinary governance. Build immune-toxicity readiness (hepatitis/colitis algorithms) into ERAS pathways; calibrate peri-operative steroids to avoid blunting IO priming when alternatives exist [83, 84].

### What negative trials teach Us

The LEAP-017 result—no statistically significant OS improvement of lenvatinib + pembrolizumab over SOC—highlights several design lessons [53]:

(i) Histology alone is not enough—stratify/enrich by metastatic pattern (liver vs. extrahepatic) and inflammatory gene programs [47, 85];
(ii) Align endpoints with biology—incorporate ctDNA clearance, depth/duration of response, treatment-free survival [14, 86];
(iii) Optimize sequence/dose—leverage the vascular-normalization window and RT fractionation to maximize immunogenicity [87, 88];
(iv) Embed window-of-opportunity cohorts to mechanistically validate “heating” beyond radiology [89, 90].

### Practical implications for today’s clinic

Outside trials: There is no broad guideline endorsement for IO combinations in unselected MSS mCRC at present [91].

Within trials (priority scenarios):

Non-liver-dominant refractory MSS mCRC → botensilimab + balstilimab or VEGFR-IO-based arms with translational cores [58, 74].

Up-front fit patients → intensification frameworks modeled on AtezoTRIBE (chemo + bevacizumab ± PD-L1) with explicit discussion of investigational status [52].

Oligometastatic disease → RT/ablation + ICI protocols that test dose/sequence and capture paired tissue + ctDNA serials [55, 56, 92].

## Data Availability

No datasets were generated or analysed during the current study.

## Abbreviations

ADCC: Antibody-dependent cellular cytotoxicity
AESI: Adverse event of special interest
CAF: Cancer-associated fibroblast
CRT: Chemoradiotherapy
ctDNA: Circulating tumor DNA
DCR: Disease control rate
DOR: Duration of response
dMMR: Mismatch-repair deficient
EGFR: epidermal growth factor receptor
ERAS: Enhanced recovery after surgery
ICI: Immune checkpoint inhibitor
IFN: Interferon
IO: Immuno-oncology
LARC: Locally advanced rectal cancer
mCRC: Metastatic colorectal cancer
MSI-H: Microsatellite instability-high
MSS: Microsatellite stable
ORR: Objective response rate
OS: Overall survival
PD-(L) 1: Programmed cell death (ligand) 1
PFS: Progression-free survival
RAS-WT: RAS wild-type
RIN: Regorafenib + ipilimumab + nivolumab
RP2D: Recommended phase-2 dose
RT: Radiotherapy
SABR: Stereotactic ablative radiotherapy
SOC: Standard of care
TKI: Tyrosine kinase inhibitor
TIL: Tumor-infiltrating lymphocyte
TME: Tumor microenvironment
TNT: Total neoadjuvant therapy
TFS: Treatment-free survival
VEGF/R: Vascular endothelial growth factor/(receptor)
